# Effectiveness and safety of switching biologics after secukinumab treatment failure in moderate-to-severe plaque psoriasis: a retrospective cohort study

**DOI:** 10.3389/fmed.2026.1866311

**Published:** 2026-07-06

**Authors:** Zhongyu Zhang, Bin Zhang, Yating Wen, Qian Hu, Wenjun Jiang, Jia Zhuo, Guangying Luo, Yuyi Wang

**Affiliations:** 1 Department of Dermatology, The First Affiliated Hospital of Chongqing College of Traditional Chinese Medicine, Chongqing, China; 2 Department of Dermatology, Chongqing Hospital of Traditional Chinese Medicine, Chongqing, China; 3 Chongqing Key Laboratory of Integrative Dermatology Research, Chongqing, China; 4 Chongqing Clinical Research Center for Dermatology, Chongqing, China

**Keywords:** biologic switching, psoriasis, real-world evidence, secukinumab, treatment failure

## Abstract

**Background:**

Biologic switching is common in clinical practice for patients with moderate-to-severe plaque psoriasis who fail secukinumab therapy. There is limited real-world evidence comparing different switching strategies, especially within-class vs. across-class approaches.

**Objective:**

To evaluate the effectiveness and safety of switching to ixekizumab, guselkumab, or ustekinumab in patients with moderate-to-severe plaque psoriasis who failed secukinumab.

**Methods:**

In this retrospective cohort study, 59 patients who switched from secukinumab to ixekizumab (*n* = 21), guselkumab (*n* = 32), or ustekinumab (*n* = 6) between January 2021 and June 2025 were analyzed. Primary endpoints were Psoriasis Area and Severity Index (PASI) 75, PASI 90, and PASI 100 response rates at weeks 12, 24, and 52. Subgroup analyses were performed by reason for switching (primary failure vs. secondary failure). Missing data were handled using non-responder imputation (NRI).

**Results:**

Ixekizumab demonstrated higher PASI 75 response rates at week 12 compared with guselkumab and ustekinumab (66.7% vs. 28.1% vs. 16.7%, *P* = 0.010). In the secondary failure subgroup, ixekizumab showed a numerical advantage in PASI 75 response at all time points that did not reach statistical significance. In the primary failure subgroup, the difference in effectiveness between ixekizumab and guselkumab did not reach statistical significance in the limited sample of this study. Adverse event rates were 28.6%, 12.5%, and 33.3% for ixekizumab, guselkumab, and ustekinumab groups, respectively, with no significant between-group differences (*P* = 0.281). All adverse events were mild and did not require treatment discontinuation.

**Conclusions:**

In patients with moderate-to-severe plaque psoriasis who fail secukinumab, switching to ixekizumab may offer advantages in short- to mid-term effectiveness, particularly in those with secondary failure. Guselkumab offers gradual but sustained improvement. All three agents demonstrate favorable safety profiles. The choice of switching strategy should integrate failure type, patient preference, and drug-specific characteristics.

## 1 Introduction

Psoriasis is a chronic, immune-mediated inflammatory skin disease affecting approximately 2%−3% of the global population (1). The introduction of biologics has fundamentally transformed the management of moderate-to-severe plaque psoriasis, with substantial improvements in clinical outcomes and quality of life (2).

Secukinumab, the first approved anti-interleukin (IL)-17A monoclonal antibody, received approval from the US Food and Drug Administration (FDA) and the European Medicines Agency (EMA) in 2015 for moderate-to-severe plaque psoriasis (3). Phase III clinical trials demonstrated PASI 75 response rates of 77%−82% and PASI 90 response rates of 54%−59% at week 12 (4, 5). Head-to-head studies further confirmed secukinumab's superiority over etanercept and ustekinumab (6), and 5-year follow-up data confirmed a stable long-term safety profile (7).

Despite these favorable outcomes, a proportion of patients experience primary or secondary treatment failure, drug-related adverse events, or personal reasons necessitating biologic switching (8). Optimal selection of subsequent therapy remains an important yet unresolved clinical question. Although several studies have examined outcomes after biologic switching, most focus on specific switching combinations or drug classes (9, 10). Comparative real-world data on switching from secukinumab to alternative biologics spanning different mechanism classes are limited, including within-class IL-17 switching to ixekizumab, IL-23 inhibition with guselkumab, and dual IL-12/23 inhibition with ustekinumab. Furthermore, the impact of failure type (primary vs. secondary) on subsequent treatment response has not been adequately characterized.

Therefore, this retrospective cohort study aimed to evaluate the effectiveness and safety of switching to ixekizumab, guselkumab, or ustekinumab after secukinumab failure in patients with moderate-to-severe plaque psoriasis, and to investigate the influence of failure type on treatment outcomes, providing real-world evidence to guide clinical decision-making.

## 2 Methods

### 2.1 Study design and participants

This was a retrospective cohort study. Consecutive patients with moderate-to-severe plaque psoriasis who were treated at the Department of Dermatology of our institution between January 2021 and June 2025 and who switched from secukinumab to ixekizumab, guselkumab, or ustekinumab due to various reasons were identified from the electronic medical record system.

Inclusion criteria: (1) diagnosis of moderate-to-severe plaque psoriasis according to the Chinese Guidelines for Psoriasis Diagnosis and Treatment (11), defined as body surface area (BSA) >3%; (2) receipt of secukinumab with subsequent switching due to inadequate response or adverse events; and (3) continued treatment and follow-up for ≥12 weeks after switching.

Exclusion criteria: (1) other psoriasis subtypes (e.g., pustular, erythrodermic); (2) severe systemic comorbidities contraindicating biologic use (active malignancy, uncontrolled cardiovascular disease, or severe hepatic or renal impairment); (3) concomitant systemic immunosuppressive therapy or phototherapy; and (4) pregnancy or lactation.

### 2.2 Data collection

The following data were extracted from medical records: (1) baseline demographics: age, sex, BMI, disease duration, comorbidities; (2) baseline disease severity: PASI score, BSA; (3) secukinumab treatment history and reason for switching; (4) post-switch biologic type and dosing records; and (5) follow-up data: PASI scores and adverse event records.

### 2.3 Definitions

Primary failure was defined as failure to achieve a PASI 50 response after the induction phase (first 12 weeks) of secukinumab (12). Secondary failure was defined as initial response to secukinumab (PASI 75 or ≥75% improvement from baseline) followed by gradual loss of effectiveness during maintenance therapy, with PASI score increasing ≥50% from the best response or loss of PASI 50 response (12). Patients who switched due to adverse events (*n* = 4) or patient preference (*n* = 2) were not classified into either failure category; they were included only in the safety analysis and excluded from the primary efficacy subgroup comparisons to reduce heterogeneity bias.

### 2.4 Treatment regimens

After secukinumab failure, patients were switched to one of the following biologics based on clinical judgment and patient preference, without a washout period:

(1) Ixekizumab (Taltz^®^): 160 mg subcutaneously at weeks 0, 2, 4, 6, 8, 10, and 12 (induction), followed by 80 mg every 4 weeks (maintenance).
(2) Guselkumab (Tremfya^®^): 100 mg subcutaneously at weeks 0 and 4 (induction), followed by 100 mg every 8 weeks (maintenance).
(3) Ustekinumab (Stelara^®^): weight-based dosing−45 mg for patients ≤ 100 kg or 90 mg for patients >100 kg at weeks 0 and 4, then every 12 weeks (maintenance).

Concomitant topical therapy (e.g., corticosteroids, vitamin D3 analogs) was permitted as adjunctive treatment. Systemic immunosuppressants and phototherapy were prohibited.

### 2.5 Effectiveness outcomes

The effectiveness endpoints were PASI 75, PASI 90, and PASI 100 response rates (defined as ≥75%, ≥90%, and 100% improvement from baseline PASI, respectively) at weeks 12, 24, and 52.

### 2.6 Safety outcomes

All adverse events (AEs) occurring during the treatment period were recorded, including infections, allergic reactions, injection site reactions, hepatic/renal function abnormalities, and hematological abnormalities.

### 2.7 Statistical analysis

Statistical analyses were performed using R software version 4.5.2. Normally distributed continuous variables are presented as mean ± standard deviation and compared using *t*-tests; non-normally distributed variables are presented as median (interquartile range) [M (Q1, Q3)] and compared using the Wilcoxon rank-sum test. Categorical variables are expressed as frequencies and percentages (%) and compared using chi-square tests or Fisher's exact tests as appropriate.

Subgroup analyses stratified by switching reason (primary vs. secondary failure) were performed to compare treatment effectiveness among groups; patients who switched due to adverse events or personal choice (*n* = 6) were excluded from efficacy subgroup comparisons. Missing outcome data were handled using non-responder imputation (NRI). Statistical significance was set at *P* < 0.05 (two-tailed). This study was a retrospective exploratory study without prospective sample size calculation; given the current sample sizes, negative results should be presented descriptively and should not be interpreted as evidence of equivalent efficacy.

The study was approved by the Ethics Review Committee of Chongqing Hospital of Traditional Chinese Medicine (ethics number: 2025-ky-55). This study was conducted in accordance with the Declaration of Helsinki. Patient data were de-identified prior to analysis.

## 3 Results

### 3.1 Patient characteristics and switching reasons

A total of 59 patients were enrolled. The cohort was predominantly male (71.2%), with a mean age of 49.5 ± 15.5 years. Reasons for switching included: secondary failure (*n* = 39, 66.1%), primary failure (*n* = 14, 23.7%), adverse events (*n* = 4, 6.8%), and patient preference (*n* = 2, 3.4%). Subgroup efficacy analyses (Tables 1 extbfand 2) were based solely on patients with skin efficacy failure (primary failure + secondary failure, *n* = 53); the 6 patients who switched due to adverse events or personal choice were excluded from subgroup analyses to reduce heterogeneity bias affecting efficacy interpretation; these 6 patients were included only in the safety analysis (Table 3). Baseline characteristics stratified by switching reason are presented in Table 4.

**Table 1:** Effectiveness comparison in the secondary failure subgroup.

Outcome	Ixekizumab (*n* = 14)	Guselkumab (*n* = 21)	*P* value
Week 12			
PASI 75 [*n* (%)]	8 (57.1%)	5 (23.8%)	0.075
PASI 90 [*n* (%)]	4 (28.6%)	5 (23.8%)	1.000
PASI 100 [*n* 15.6-7.2,-1.3242pt(%)]	2 (14.3%)	3 (14.3%)	1.000
Week 24			
PASI 75 [*n* (%)]	8 (57.1%)	8 (38.1%)	0.317
PASI 90 [*n* (%)]	4 (28.6%)	5 (23.8%)	1.000
PASI 100 [*n* 15.6-7.2,-1.3242pt(%)]	2 (14.3%)	3 (14.3%)	1.000
Week 52			
PASI 75 [*n* (%)]	7 (50.0%)	8 (38.1%)	0.511
PASI 90 [*n* (%)]	6 (42.9%)	5 (23.8%)	0.283
PASI 100 [*n* (%)]	4 (28.6%)	4 (19.0%)	0.685

PASI, psoriasis area and severity index; PASI 75/90/100, ≥75%/≥90%/100% improvement in PASI from baseline.

**Table 2:** Effectiveness comparison in the primary failure subgroup.

Outcome	Ixekizumab (*n* = 6)	Guselkumab (*n* = 6)	*P* value
Week 12			
PASI 75 [*n* (%)]	4 (66.7%)	3 (50.0%)	1.000
PASI 90 [*n* (%)]	2 (33.3%)	2 (33.3%)	1.000
PASI 100 [*n* 15.6-7.2,-1.3242pt(%)]	2 (33.3%)	1 (16.7%)	1.000
Week 24			
PASI 75 [*n* (%)]	5 (83.3%)	3 (50.0%)	0.535
PASI 90 [*n* (%)]	2 (33.3%)	2 (33.3%)	1.000
PASI 100 [*n* 15.6-7.2,-1.3242pt(%)]	2 (33.3%)	2 (33.3%)	1.000
Week 52			
PASI 75 [*n* (%)]	4 (66.7%)	5 (83.3%)	0.242
PASI 90 [*n* (%)]	2 (33.3%)	2 (33.3%)	1.000
PASI 100 [*n* (%)]	1 (16.7%)	2 (33.3%)	1.000

PASI, psoriasis area and severity index; PASI 75/90/100, ≥75%/≥90%/100% improvement in PASI from baseline.

**Table 3:** Adverse events by treatment group.

Adverse event	Ixekizumab (*n* = 21)	Guselkumab (*n* = 32)	Ustekinumab (*n* = 6)	Total (*n* = 59)	*P* value
**Patients with any AE [*****n*** **(%)]**	**6 (28.6%)**	**4 (12.5%)**	**2 (33.3%)**	**12 (20.3%)**	**0.281**
Urticaria	1 (4.8%)	1 (3.1%)	1 (16.7%)	3 (5.1%)	0.473
Eczema	3 (14.3%)	1 (3.1%)	0 (0%)	4 (6.8%)	0.680
Injection site reaction	3 (14.3%)	1 (3.1%)	0 (0%)	4 (6.8%)	0.251
Rhinorrhea	0 (0%)	1 (3.1%)	0 (0%)	1 (1.7%)	1.000
Pharyngalgia	0 (0%)	1 (3.1%)	0 (0%)	1 (1.7%)	1.000
Ocular pruritus	0 (0%)	1 (3.1%)	0 (0%)	1 (1.7%)	1.000
Generalized pruritus	1 (4.8%)	0 (0%)	1 (16.7%)	2 (3.4%)	0.082
Total AE events reported	8	6	2	16	—
Mean AE events per patient	0.38	0.19	0.33	0.27	—

AE, adverse event. All AEs were Grade 1 (mild). Mean events per patient = total events/number of patients in group. Bold values indicate the overall adverse event summary variables, including patients with any adverse event, total adverse event events reported, and mean adverse event events per patient.

**Table 4:** Baseline characteristics of patients stratified by switching reason.

Characteristic	Overall (*n* = 59)	Primary failure (*n* = 14)	Secondary failure (*n* = 39)	AEs (*n* = 4)	Patient choice (*n* = 2)
Age [years, M (Q1, Q3)]	51.0 (37.5, 60.0)	51.0 (38.0, 62.3)	52.0 (39.5, 59.5)	36.5 (32.0, 44.8)	45.0 (38.5, 51.5)
Male sex [*n* (%)]	42 (71.2%)	7 (50.0%)	30 (76.9%)	4 (100%)	1 (50.0%)
BMI [kg/m^2^, M (Q1, Q3)]	25.2 (23.2, 27.7)	27.9 (25.7, 30.4)	24.7 (22.6, 26.3)	23.7 (22.7, 24.5)	23.6 (21.1, 26.0)
Current smoker [*n* (%)]	26 (44.1%)	5 (35.7%)	18 (46.2%)	3 (75.0%)	0 (0%)
Alcohol use [*n* (%)]	14 (23.7%)	2 (14.3%)	11 (28.2%)	1 (25.0%)	0 (0%)
Metabolic syndrome [*n* (%)]	24 (40.7%)	7 (50.0%)	16 (41.0%)	0 (0%)	1 (50.0%)
Psoriatic arthritis [*n* (%)]	9 (15.3%)	3 (21.4%)	5 (12.8%)	0 (0%)	1 (50.0%)
Disease duration [years, M (Q1, Q3)]	11.0 (6.0, 22.0)	13.0 (5.8, 23.8)	11.0 (6.0, 24.5)	10.5 (7.0, 14.8)	5.3 (2.9, 7.6)
Age at onset [years, M (Q1, Q3)]	32.0 (22.5, 45.0)	33.5 (24.3, 45.3)	32.0 (21.0, 45.0)	26.0 (23.5, 31.5)	40.0 (36.0, 44.0)
Baseline PASI [M (Q1, Q3)]	13.0 (8.0, 22.3)	8.2 (5.3, 20.2)	14.7 (8.8, 22.9)	13.1 (12.1, 15.7)	13.1 (8.7, 17.4)

BMI, body mass index; M, median; PASI, psoriasis area and severity index; Q1, first quartile; Q3, third quartile.

### 3.2 Overall treatment effectiveness

PASI 75 response rates for the ixekizumab group were 66.7%, 66.7%, and 57.1% at weeks 12, 24, and 52, respectively, remaining significantly higher than those for guselkumab (28.1%, 40.6%, and 40.6%) and ustekinumab (16.7%, 16.7%, and 16.7%) at week 12 (*P* = 0.010). The guselkumab group demonstrated progressive improvement from week 12 to week 52, while the ustekinumab group maintained relatively stable but low response rates throughout.

For PASI 90, the ixekizumab group showed continuous improvement from 33.3% at week 12 to 42.9% at week 52. The guselkumab group reached 25.0% and 28.1% at weeks 12 and 24, but declined to 21.9% at week 52. The ustekinumab group achieved no PASI 90 responses at any time point.

**Figure 1:**
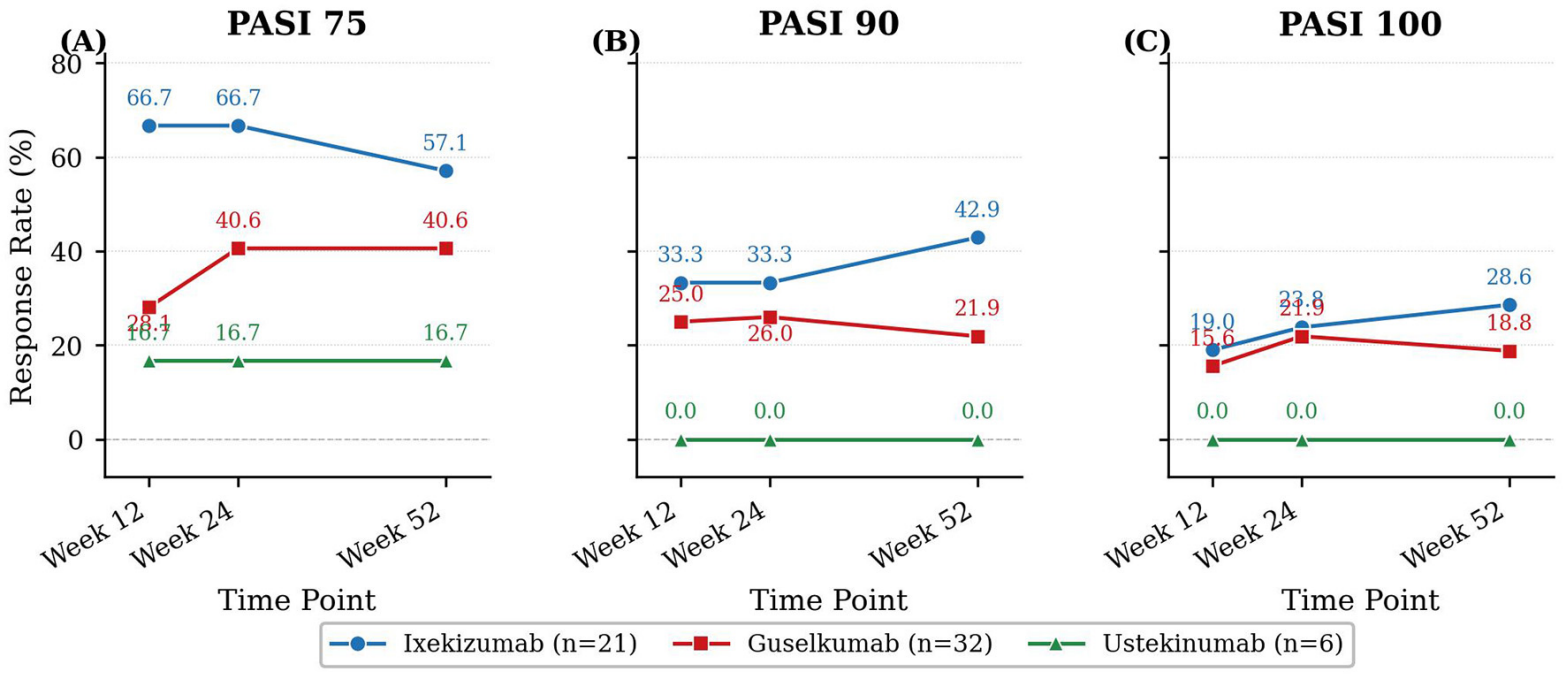
PASI response rates over 52 weeks by treatment group. **(A)** PASI 75, **(B)** PASI 90, and **(C)** PASI 100 response rates at Weeks 12, 24, and 52 among patients receiving ixekizumab (*n* = 21), guselkumab (*n* = 32), or ustekinumab (*n* = 6). PASI, psoriasis area and severity index; PASI 75/90/100, ≥75%/≥90%/100% improvement in PASI from baseline.

Regarding PASI 100 (complete clearance), ixekizumab response rates increased from 19.0% at week 12 to 28.6% at week 52. Guselkumab rates rose from 15.6% at week 12 to 21.9% at week 24, with a slight decline to 18.8% at week 52. No patients in the ustekinumab group achieved complete skin clearance. Complete effectiveness data are presented in Table 5, and response rate trends over 52 weeks are illustrated in Figure 1.

**Table 5:** Treatment effectiveness comparison among the three biologic groups.

Outcome	Ixekizumab (*n* = 21)	Guselkumab (*n* = 32)	Ustekinumab (*n* = 6)	*P* value
Week				
PASI 75 [*n* (%)]	14 (66.7%)	9 (28.1%)	1 (16.7%)	0.010^*^
PASI 90 [*n* (%)]	7 (33.3%)	8 (25.0%)	0 (0%)	0.290
15.6-7.2,-1.3498ptPASI 100 [*n* (%)]	4 (19.0%)	5 (15.6%)	0 (0%)	0.769
Week 24				
PASI 75 [*n* (%)]	14 (66.7%)	13 (40.6%)	1 (16.7%)	0.060
PASI 90 [*n* (%)]	7 (33.3%)	9 (28.1%)	0 (0%)	0.301
15.6-7.2,-1.3498ptPASI 100 [*n* (%)]	5 (23.8%)	7 (21.9%)	0 (0%)	0.646
Week 52				
PASI 75 [*n* (%)]	12 (57.1%)	13 (40.6%)	1 (16.7%)	0.184
PASI 90 [*n* (%)]	9 (42.9%)	7 (21.9%)	0 (0%)	0.092
PASI 100 [*n* (%)]	6 (28.6%)	6 (18.8%)	0 (0%)	0.244

^*^P < 0.05. PASI, psoriasis area and severity index; PASI 75/90/100, ≥75%/≥90%/100% improvement in PASI from baseline.

### 3.3 Subgroup analysis by failure type

#### 3.3.1 Secondary failure subgroup

The secondary failure subgroup included 39 patients: ixekizumab (*n* = 14), guselkumab (*n* = 21), and ustekinumab (*n* = 4). Due to the small ustekinumab group size, formal statistical comparisons were limited to ixekizumab vs. guselkumab.

At week 12, the ixekizumab group showed a numerically higher PASI 75 response rate than guselkumab (57.1% vs. 23.8%, *P* = 0.075). At week 24, ixekizumab maintained 57.1% vs. 38.1% for guselkumab (*P* = 0.317). At week 52, PASI 75, PASI 90, and PASI 100 response rates were 50.0%, 42.9%, and 28.6% for ixekizumab vs. 38.1%, 23.8%, and 19.0% for guselkumab, respectively, with no statistically significant between-group differences. Data are presented in Table 1.

#### 3.3.2 Primary failure subgroup

The primary failure subgroup included 14 patients: ixekizumab (*n* = 6), guselkumab (*n* = 6), and ustekinumab (*n* = 2). Statistical comparisons were limited to ixekizumab vs. guselkumab. In the limited sample of this study, the differences in effectiveness between the two groups did not reach statistical significance at all time points (all *P* > 0.05); however, given the extremely small sample size, clinically meaningful differences cannot be ruled out. PASI 75 response rates increased over time in both groups: the guselkumab group achieved a maximum PASI 75 response of 83.3% at week 52, while ixekizumab peaked at 83.3% at week 24 before declining slightly to 66.7% at week 52. PASI 90 and PASI 100 response rates were relatively low and showed no clear time-dependent trend. Data are shown in Table 2.

### 3.4 Safety

A total of 16 AE events were reported across 12 patients, with an overall incidence of 20.3% (12/59). AE rates were 28.6% (6/21), 12.5% (4/32), and 33.3% (2/6) for ixekizumab, guselkumab, and ustekinumab groups, respectively, with no significant between-group difference (*P* = 0.281). All AEs were Grade 1 (mild) and did not require treatment discontinuation. The most common AEs were injection site reactions (*n* = 4, 6.8%), urticaria (*n* = 3, 5.1%), and eczema (*n* = 4, 6.8%). Mean AE events per patient were 0.38, 0.19, and 0.33 for the ixekizumab, guselkumab, and ustekinumab groups, respectively. No serious infections, candidiasis, or inflammatory bowel disease were observed. Adverse event data are summarized in Table 3.

## 4 Discussion

This study systematically evaluated the effectiveness and safety of switching to ixekizumab, guselkumab, or ustekinumab in patients with moderate-to-severe plaque psoriasis following secukinumab failure. Our real-world data demonstrate that in the present cohort, within-class switching to ixekizumab showed numerically higher PASI 75 response rates at week 12, and all three regimens demonstrated favorable safety profiles.

Ixekizumab achieved a PASI 75 response rate of 66.7% at week 12, significantly higher than guselkumab (28.1%) and ustekinumab (16.7%). These findings are consistent with previous reports. Georgakopoulos et al. (13) reported a PASI 75 response rate of 69% at week 12 in secukinumab non-responders switched to ixekizumab in a multicenter retrospective study. Sherman et al. (14) similarly reported a PASI 90 response rate of 71.4% at week 12 in 25 patients switched from secukinumab to ixekizumab. Collectively, these data support the feasibility and effectiveness of within-class IL-17A switching.

The higher PASI 75 response rate observed in the ixekizumab group at week 12 in this study suggests that some patients who fail secukinumab may retain responsiveness to another IL-17A inhibitor within the same class. This may be related to differences between the two agents in molecular structure (humanized IgG4 vs. fully human IgG1), IL-17A binding epitope, dosing frequency, and pharmacokinetic properties (15, 16). Bernardini et al. (17) reported that 91% of patients achieved PASI 90 after switching from ixekizumab to secukinumab, further supporting the concept of bidirectional intra-class effectiveness.

Patients switching to guselkumab exhibited progressive improvement, with PASI 75 response rates rising from 28.1% at week 12 to 40.6% at week 52. This pattern of delayed but sustained response is consistent with the mechanism of IL-23 inhibition: IL-23 is a pivotal cytokine for Th17 cell differentiation and survival, and blocking this pathway upstream of IL-17 may require more time to deplete the established Th17 cell pool (18). Megna et al. (19) reported markedly higher PASI 75 response rates (93.2%) in 44 anti-IL-17-experienced patients switched to guselkumab over 52 weeks—differences that likely reflect heterogeneous baseline disease severity and prior biologic exposure. Warren et al. (9) similarly demonstrated robust responses in patients switched from secukinumab or ixekizumab to risankizumab (another IL-23p19 inhibitor) in the phase 3b aIMM study, with 57.4% achieving sPGA 0/1 at week 16 and 67.6% at week 52.

Our findings warrant interpretation against head-to-head comparative evidence. In the pivotal IXORA-R randomized trial of biologic-naïve patients, ixekizumab produced faster and significantly higher complete clearance than guselkumab at week 12 (PASI 100, 41.3% vs. 24.9%; *P* < 0.001), whereas the two agents became comparable by week 24 (PASI 100, 49.8% vs. 52.3%) (27, 28). The trajectory in our cohort—an early advantage for ixekizumab followed by progressive, sustained improvement with guselkumab—mirrors this established difference in speed of onset. Some subgroup observations nonetheless diverge from the overall ixekizumab superiority reported in treatment-naïve populations; most notably, within the primary failure subgroup guselkumab numerically exceeded ixekizumab in PASI 75 response at week 52 (83.3% vs. 66.7%). This apparent discrepancy most plausibly reflects population differences rather than a true reversal of efficacy: our patients had all failed prior IL-17A inhibition—a context absent from head-to-head trials—and primary IL-17A failure may attenuate the response to within-class IL-17A switching while favoring upstream IL-23 blockade. The very small subgroup sizes (*n* = 6 per arm) further render these differences statistically unstable and hypothesis-generating only. Rather than contradicting existing data, our results suggest that the relative benefit of ixekizumab vs. guselkumab is modified by prior treatment history and failure type.

In the limited sample of this study, the ustekinumab group showed numerically lower response rates than the other two groups (PASI 75: 16.7% at all time points), which differs from the report by Ruggiero et al. (20), who found 87.5% PASI 75 and 50% PASI 90 responses at week 48 in 21 patients switched from secukinumab. Given the severely inadequate sample size in our ustekinumab group (*n* = 6), the above values are provided for descriptive reference only, lack inferential value, and should not be used to conclude that ustekinumab is less effective than other regimens in this setting. Potential explanations include selection bias (toward refractory cases) and pharmacological differences between the IL-12/23p40 inhibitor and newer IL-23p19-selective agents (21); however, larger studies are needed for verification.

Subgroup analyses revealed clinically meaningful differences between primary and secondary failure patients. Among secondary failure patients, ixekizumab consistently demonstrated numerically higher PASI 75 rates at all time points, although significance was not reached—likely due to limited sample size. This finding is conceptually coherent: secondary failure implies initial responsiveness to IL-17A inhibition, and within-class switching to another IL-17A inhibitor may recapture disease control. In contrast, primary failure may indicate IL-17A-independent pathogenesis, theoretically favoring upstream blockade with IL-23 inhibitors (22). However, in our primary failure subgroup, the differences in effectiveness between the two agents did not reach statistical significance in the limited sample, which may reflect the complex immunopathology and inter-individual heterogeneity of psoriasis, and may also be related to the insufficient sample size (*n* = 6 vs. *n* = 6) limiting statistical power; this should not be interpreted as evidence of equivalent efficacy between the two groups.

The prognostic significance of failure type has been reported by Torres et al. (8), who showed that primary failure was associated with significantly shorter drug survival in a multicenter cohort of 1,580 psoriasis patients. Our data similarly suggest that primary failure portends a challenging therapeutic course, potentially requiring combination strategies or novel therapeutic targets.

Two contextual factors further temper our findings. First, efficacy was assessed using the skin-focused PASI, which does not capture multi-domain involvement; special sites—nail, scalp, palmoplantar, and genital disease—influence biologic selection, and agents differ across domains (e.g., ixekizumab and secukinumab perform well for nail disease, guselkumab for scalp disease), so the weight of these factors in real-world switching may have been underestimated. Second, psoriatic arthritis (PsA) was present in nine patients (15.3%; ixekizumab *n* = 3, guselkumab *n* = 5, ustekinumab *n* = 1), in all of whom secukinumab failure was driven primarily by inadequate skin response. Biologic switching in PsA carries complexity beyond skin outcomes: real-world data indicate that switching mechanism of action rather than within-class cycling improves treatment retention after TNF inhibitor failure (24, 25), and IL-23 inhibitors retain meaningful efficacy in biologic-experienced peripheral PsA (26). The limited number of PsA patients (*n* = 9) precluded subtype-specific analysis. Future studies should incorporate composite musculoskeletal endpoints (e.g., ACR20/50, DAPSA, LEI) alongside multi-domain skin scoring to evaluate each switching strategy more comprehensively.

All three biologics demonstrated excellent tolerability, with mild, grade 1 AEs only and no treatment discontinuations. The safety profile is consistent with established literature (4, 23). Notably, no serious infections, candidiasis, or inflammatory bowel disease were observed—AEs characteristically associated with IL-17 inhibitors. This may reflect the relatively short follow-up duration (up to 52 weeks) and limited sample size; longer-term prospective data are warranted.

Several limitations should be acknowledged. As a retrospective study, selection and information bias are inherent. The small sample sizes—particularly the ustekinumab group (*n* = 6) and the primary failure subgroup (*n* = 14)—severely limit statistical power and generalizability; as an exploratory study without prospective sample-size calculation, negative results should be interpreted descriptively rather than as evidence of equivalent efficacy. Special site involvement (nail, scalp, palmoplantar, genital), patient-reported outcomes (e.g., DLQI), and immunological biomarkers were not systematically captured, limiting multi-domain and mechanistic insight; classification of switching reason relied partly on clinical documentation and may carry some subjectivity. The limited number of PsA patients (*n* = 9) precluded subtype-specific analysis, and follow-up of up to 52 weeks precludes assessment of long-term drug survival. Finally, newer IL-23p19 inhibitors (e.g., risankizumab, tildrakizumab) were not included. Future studies should adopt standardized multi-domain failure definitions and incorporate disease scoring across skin and musculoskeletal domains.

In conclusion, for patients with moderate-to-severe plaque psoriasis who fail secukinumab, in the limited sample of this study, switching to ixekizumab showed numerically higher PASI 75 response rates at week 12, particularly in secondary failure cases, and this result may be related to differences between the two agents in molecular structure, dosing frequency, and IL-17A binding characteristics. Guselkumab, while slower in onset, offers sustained improvement and may be suitable for patients who can tolerate a delayed onset of action. Data for the ustekinumab group (*n* = 6) are provided for descriptive reference only due to insufficient sample size and should not be used for inferential conclusions. All three agents demonstrated favorable safety profiles. Future large-scale, prospective, long-term studies are needed to further optimize biologic switching strategies and identify predictive biomarkers for personalized treatment selection, and should incorporate multi-domain disease scoring to comprehensively evaluate the benefits of each switching regimen.

## Data Availability

The raw data supporting the conclusions of this article will be made available by the authors, without undue reservation.
